# Body dynamics of gait affect value-based decisions

**DOI:** 10.1038/s41598-021-91285-1

**Published:** 2021-06-04

**Authors:** Eric Grießbach, Francesca Incagli, Oliver Herbort, Rouwen Cañal-Bruland

**Affiliations:** 1 Department for the Psychology of Human Movement and Sport, Friedrich Schiller University Jena, Jena, Germany; 2 Department of Psychology, Julius-Maximilians-Universität Würzburg, Würzburg, Germany

**Keywords:** Psychology, Human behaviour, Motor control, Reward

## Abstract

Choosing among different options typically entails weighing their anticipated costs and benefits. Previous research has predominantly focused on situations, where the costs and benefits of choices are known before an action is effectuated. Yet many decisions in daily life are made on the fly, for instance, making a snack choice while walking through the grocery store. Notably, the costs of actions change dynamically while moving. Therefore, in this study we examined whether the concurrent action dynamics of gait form part of and affect value-based decisions. In three experiments, participants had to decide which lateral (left vs. right) target (associated with different rewards) they would go to, while they were already walking. Results showed that the target choice was biased by the alternating stepping behavior, even at the expense of receiving less reward. These findings provide evidence that whole-body action dynamics affect value-based decisions.

## Introduction

Imagine yourself walking through the grocery store. While walking down the aisle in the candy section, you start having an appetite for candy. To your left you see your favorite liquorice. Somewhat closer to your right you see your favorite fruit gums. Which snack will you go for? Value-based decision-making is often considered to be a cognitive weighing process between costs and benefits^1,2^. In this scenario, the benefit would perhaps be reflected by the caloric intake or tastiness of either of the two snacks, and the costs might include the cost of the action itself, here the physical effort it may take to walk to the liquorice, which is farther away than the fruit gums. There is empirical evidence supporting the claim that the costs of action play a significant role in decision-making^3–7^.

However, the majority of this research investigates just a snapshot of human decisions, namely situations in which choices and actions can be implemented sequentially. Per definition, in sequential decisions, cost and reward information is available before an action is initiated. Only after weighing the options, the action is executed. Theories of sequential decision-making such as good-based models^8^ and evidence accumulation models^9^ assume that costs and rewards are being weighted independently of actions. Good-based models focus on where the competition between action choices occurs^8,11^. They assume that the comparison of choice options takes place at an abstract level independent of sensorimotor representations. As such, decision and action are separate, sequentially unfolding modules. Only after reaching a decision boundary modeled as competition between abstract choice options, the decision is accomplished and implemented by a respective sensorimotor action.

Evidence accumulation models focus on a formal specification of how selection occurs^9,11^. More specifically, evidence is sampled in a sequential manner until one choice option reaches a threshold. Similar to good-based models, only afterwards an action is initiated. It follows that in these theories the flow of information is modeled in a unidirectional manner: the choice governs the action^8,9^.

According to Lepora and Pezzulo^10^, when a decision is made and only afterwards an action is initiated, by definition the action dynamics—evolving a posteriori—cannot influence the already made decision without feedback from action dynamics. Consequently, sequential decision-making theories^8,9^ cannot account for many situations in which decisions have to be made during action execution, be it in sports (e.g., when deciding whether to pass a defender on the left or right while dribbling the ball), work environments (e.g., when navigating through a construction site), or other everyday situations (e.g., when making a snack choice while walking). In such situations costs of actions change dynamically and hence may need to be continuously updated and integrated into the decision process, a process not covered by sequential decision-making models.

Therefore, alternative theoretical approaches have been proposed, including the embodied choice framework^10^ and action-based models^11–13^. These approaches do integrate dynamic action costs in decision-making. The embodied choice framework assumes bidirectional, continuous feedback between the action and the decision process^10^. This entails feedback about dynamic action costs that are continuously fed back into the decision. Action-based models propose that the degree of activation between competing actions reflects the weighing of costs and rewards, thereby arguing that action and decision processes form an inseparable unity^11–13^.

While these theoretical approaches have received support from neurophysiological studies^12,14^, there are only a handful of behavioral studies that examined the impact of concurrent action dynamics^15–18^. These studies, however, tend to report rather mixed evidence. On the one hand, Wolpert et al.^16^ showed that perceptual decisions are influenced by dynamic action costs in a reaching task. On the other hand, Michalski et al.^18^ provided evidence that in a finger tracking task the dynamic action cost was only integrated into the decision process when the demands of continuous tracking were removed. Therefore, a first open question that remains to be answered is whether dynamic action costs are integrated into behavioral decisions, and if so, whether this effect translates to whole-body movements (going beyond reaching and pointing), thereby generalizing to a broad range of ecological choices in daily situations.

A second open question concerns the time course of action cost integration. In this regard, Bakker et al.^17^ provided initial evidence that when dynamic action costs are integrated into a reaching task, this is based on the immediate body state rather than the anticipated body state that per definition lies in the future and are bound to change continuously. However, given that this study applied a paradigm that only included passive motions^17^, the time course of action cost integration in decisions during active movements such as when walking through the aisle of the grocery store to buy candy is yet to be determined.

**Figure 1:**
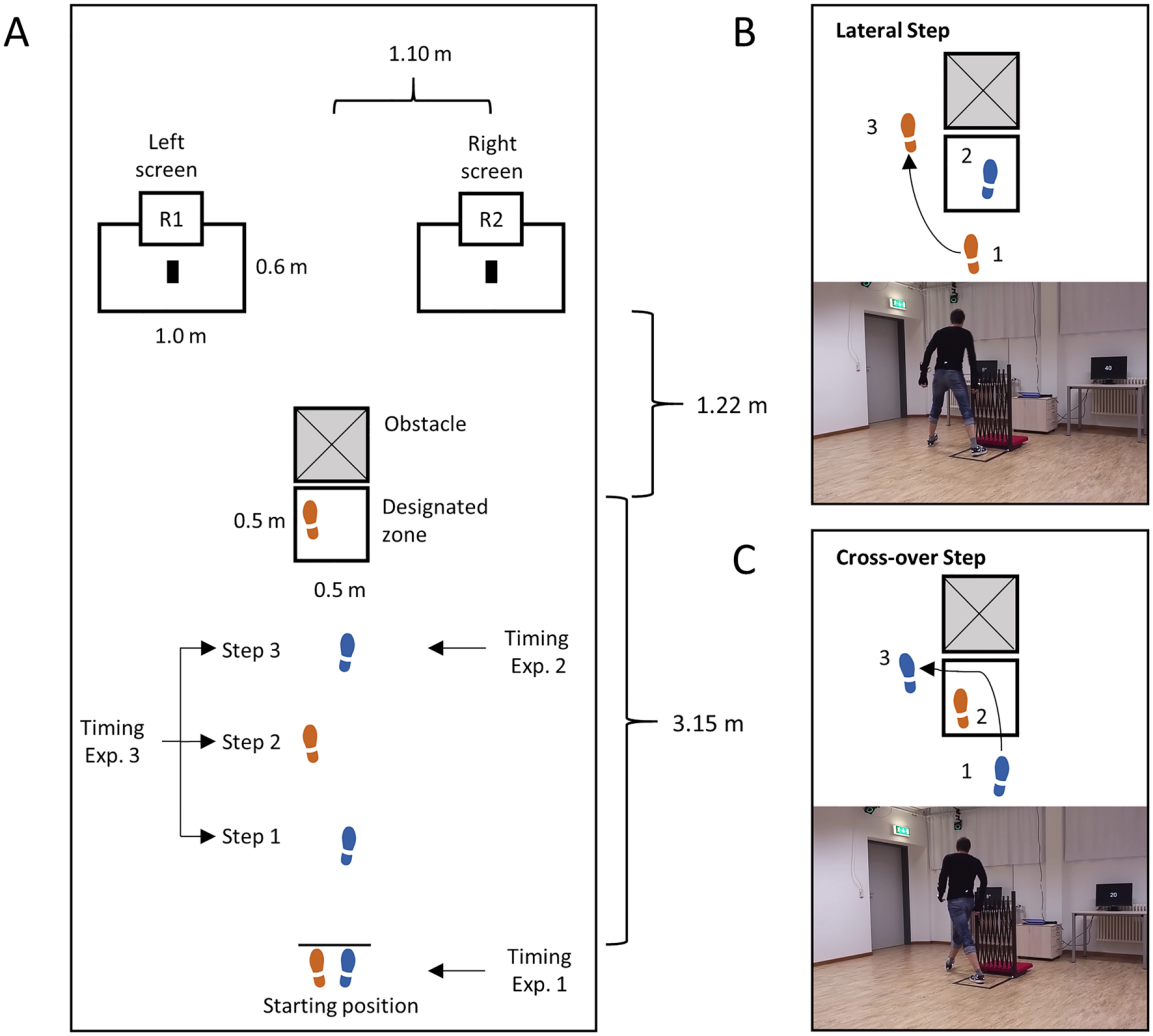
Experimental setup and exemplary stepping behavior to bypass the obstacle. (**A**) Dimensions of the experimental setup. Proportions are scaled to closely fit the real setup in this figure. Participants started a trial with the feet next to each other (Exp. 1) or a prespecified foot was placed at the starting line and the other foot was positioned behind, thereby determining the first step and stepping behavior towards the obstacle (Exp. 2 and 3, not shown in the figure). Rewards were displayed on the left and right screens before walking towards the obstacle (Exp. 1) or while walking towards the obstacle (Exp. 2 and 3). To determine the timing of the reward presentation the positions of the shoes were measured kinematically with a 3D infrared camera system in real-time and the time point of the touch-down for each step was estimated^25^. Rewards were displayed at the touch-down one step (Exp. 2) or between three to one steps (Exp. 3) before stepping into the designated zone. To get to the reward, participants were instructed to step into the designated zone before bypassing the obstacle. Participants ended a trial by touching the black rectangle on either desk with one hand. (**B**) Example for the lateral step. Here the right foot stepped into the designated zone before walking towards the left target. (**C**) Example for the cross-over step. Here the left foot stepped into the designated zone before walking towards the left target. For convenience, the left foot is displayed in orange and the right foot in blue. *R1 *reward left side, *R2 *reward right side.

To recap, if indeed dynamic changes of the body state (i.e., dynamic action costs) are part of the decision process in daily human behaviors, then the decision in the introductory example to choose between the liquorice or the fruit gums should be influenced by the concurrent stepping (i.e., walking) behavior. To test this, here we examined how walking, a complex whole-body movement, affects value-based decision-making in three experiments in which reward options appeared to the left or right side during walking (see Fig. 1). During walking the body state alternates between the left and right foot supporting the body. Based on the foot on the ground, the action costs of making a directional change vary dynamically. That is, if the left foot is currently on the ground and we intend to walk towards a target at the right, the swing leg (right) can make a lateral step towards the right. If in the same scenario, we intend to walk towards a target at the left, the right swing leg would have to make a cross-over step towards the left side (see Fig. 1). Prior work showed a preference for the lateral stepping strategy over cross-over steps when avoiding a planar obstacle on the ground^19,20^. More specifically, a cross-over step was more unstable than a lateral step because of a reduced area on the ground to stabilize the laterally swaying center of mass. When participants were free to choose a directional change towards the left or right side, participants had a higher success rate and preference to change the direction towards the side which enabled a lateral step and avoided the cross-over step^21^. This finding confirms that a directional change by making a cross-over step is costlier than a lateral step. Costlier is defined quite liberally here (i.e., is not limited to bioenergetic costs only^20,22^), denoting any difference of characteristics between actions (including e.g. stability^19^) that render one action preferable or more likely than the other.

To validate that in our walking paradigm (see Fig. 1) the cross-over step was indeed costlier than a lateral step, in Exp. 1, we examined the preference for either stepping strategy in sequential decision-making, that is, when cost and reward information was available before the first step was initiated. Knowing that in sequential decision tasks participants typically adapt their coordination pattern to assume a body state that facilitates the realization of their decision^23,24^, we predicted a preference for the lateral rather than the cross-over stepping strategy. Results confirmed this prediction.

This validation allowed us to subsequently address the two main questions highlighted above. First, based on the embodied choice framework^10^ and action-based models^11–13^, we examined whether the dynamic action costs during walking influence value-based decisions. Second, we aimed at scrutinizing the time course of such action cost integration. Given previous evidence from research on reaching tasks indicating that dynamic action costs of the immediate body state rather than the anticipated body state is integrated into the decision process^17^, in Exp. 2, we first tested whether this prediction proved robust for whole-body movements such as displayed in our walking paradigm (see Fig. 1). To this end, we presented the reward information so late that the immediate body state would necessarily dictate the subsequent lateral or cross-over step. In other words, if participants were to integrate dynamic action costs, in this condition this could be only achieved by integrating the immediate (but not anticipated) body state due to the temporal demands of the task. It follows that based on the embodied choice framework^10^ and action-based models^11–13^, in Exp. 2 we predicted that participants would be biased towards a lateral stepping strategy, even at the expense of getting lower rewards.

Because Exp. 2 did not differentiate between the integration of the dynamic action costs of the immediate vs. the anticipated body state, we conducted a third experiment. Exp. 3 allowed us to scrutinize the time course of action cost integration in value-based decisions in a more fine-grained manner. That is, we systematically manipulated three time points of displaying the reward information during walking, including earlier reward presentation conditions that gave participants more time to potentially anticipate the final body state mandating the lateral or cross-over step. We hypothesized that if it was indeed the immediate body state at the time of reward presentation (and not the anticipated body state) that affects the value-based decision, then the immediate body state would predict the final stepping direction regardless of the anticipated body state dictating a lateral step. This should hence be observable independent of congruency or incongruency between the immediate and the anticipated body state, even when resulting in lower rewards at higher action costs.

## Results

### Adaptation of stepping behavior enables a lateral step in sequential decision-making

As in the introductory grocery store example, we chose a task in which participants were walking while reward options appeared on the left or right side (see Fig. 1A). To get the reward, participants had to step with at least one foot into a designated zone in front of a central obstacle and bypass it to its left or right to walk to one of the lateral targets. As rewards different combinations of points were displayed at the left and right lateral target (e.g., 60 points left and 40 points right). The points always summed up to 100. To first assess and control whether participants would indeed prefer a lateral stepping strategy compared to a cross-over step (see Fig. 1B,C), in Exp. 1 the rewards were displayed before participants started walking. That is, cost and reward information were available before an action was initiated. Participants started a trial in a neutral position with the feet next to each other. The stepping behavior was measured kinematically by attaching reflective markers on the shoes and measuring their position with a 3D-infrared camera system (see “Methods”).

**Figure 2:**
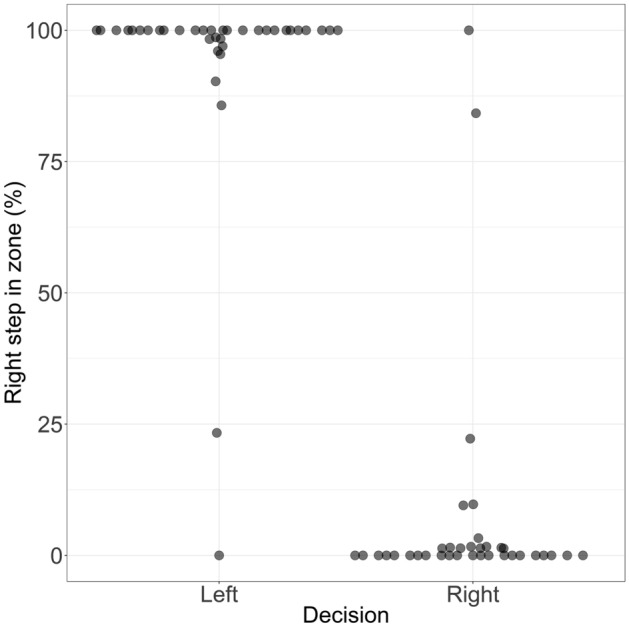
Adaptation of the step into the zone enabling a lateral step. When participants walked towards the left side, they stepped more frequently into the zone with the right foot and vice versa. This shows the preference for a lateral step over the cross-over step, thereby confirming that the cross-over step is indeed costlier. Each dot displays the probability for individual subjects of making a step with the right foot into the zone. Zero percent indicates that participants always made a left step into the zone. Dots are jittered for better visual inspection.

In the sequential decision-making task of Exp. 1, participants followed the instruction and almost always went toward the side with higher rewards (99.9%). Only when there was no reward difference, choices were more variable (see Supplementary Fig. S1A). Regarding the stepping strategy, participants adapted the final step into the zone to enable a lateral step (see Fig. 2).

Specifically, when participants walked to the reward presented at the right side, they more frequently stepped with the left foot into the designated zone and vice versa (*χ*^2^ (1) = 59.30, *p* < 0.001, OR = 0.00010, 95% CI [0.00001, 0.00020]). To examine whether participants took into account the cost information before the first step was initiated, we additionally analyzed if participants already adapted their first step based on the decision they finally effectuated. Results showed that participants indeed varied the leg to start walking with or the step length to enable a final lateral stepping strategy (see SI, Fig S2), indicating that the cost information was taken into account before action initiation.

The adaptation of the stepping strategy validated that in our walking paradigm the cross-over step was indeed costlier than a lateral step when cost and reward information was available before the first step was initiated, that is, in sequential decision-making.

Following this validation, in Exp. 2 we then tested whether the dynamic action costs of the immediate body state are integrated into the decision process (see Fig. 1) as predicted by research on reaching^17^. To this end, we presented the reward information late so that the immediate body state would inexorably dictate the subsequent lateral or cross-over step. Based on the embodied choice framework^10^ and action-based models^11–13^, we hypothesized a bias towards a lateral stepping strategy, even at the expense of receiving less rewards.

### Dynamic action costs influence immediate value-based decisions

In Exp. 2, rewards were displayed while participants were approaching and close to the obstacle. Specifically, the reward information was displayed at the kinematically estimated touch-down (first contact of the foot with the ground)^25^ one step before stepping into the designated zone. The localization of this step was determined based on Exp. 1. It typically concerned the third step which took on average 490 ms (sd = 111 ms) until the touch-down of the final step into the zone. To control the final step into the zone (dictating either a lateral or cross-over step) and its combination with the reward information (e.g., 60 points left vs. 40 points right), we manipulated the starting position (left or right leg in front, resulting in a first step with the right or left foot, respectively) randomly on each trial. Additionally, to regulate the difficulty of the task, we constrained the temporal demands of reaching the target. Based on the data of Exp. 1, we included a ‘regular walking’ condition (6 s) and a ‘time pressure’ condition (4 s). If participants integrated dynamic action costs based on the body state assumed when stepping into the zone, then we hypothesized a bias towards a lateral stepping strategy, independent of and hence even at the expense of receiving less rewards.

**Figure 3:**
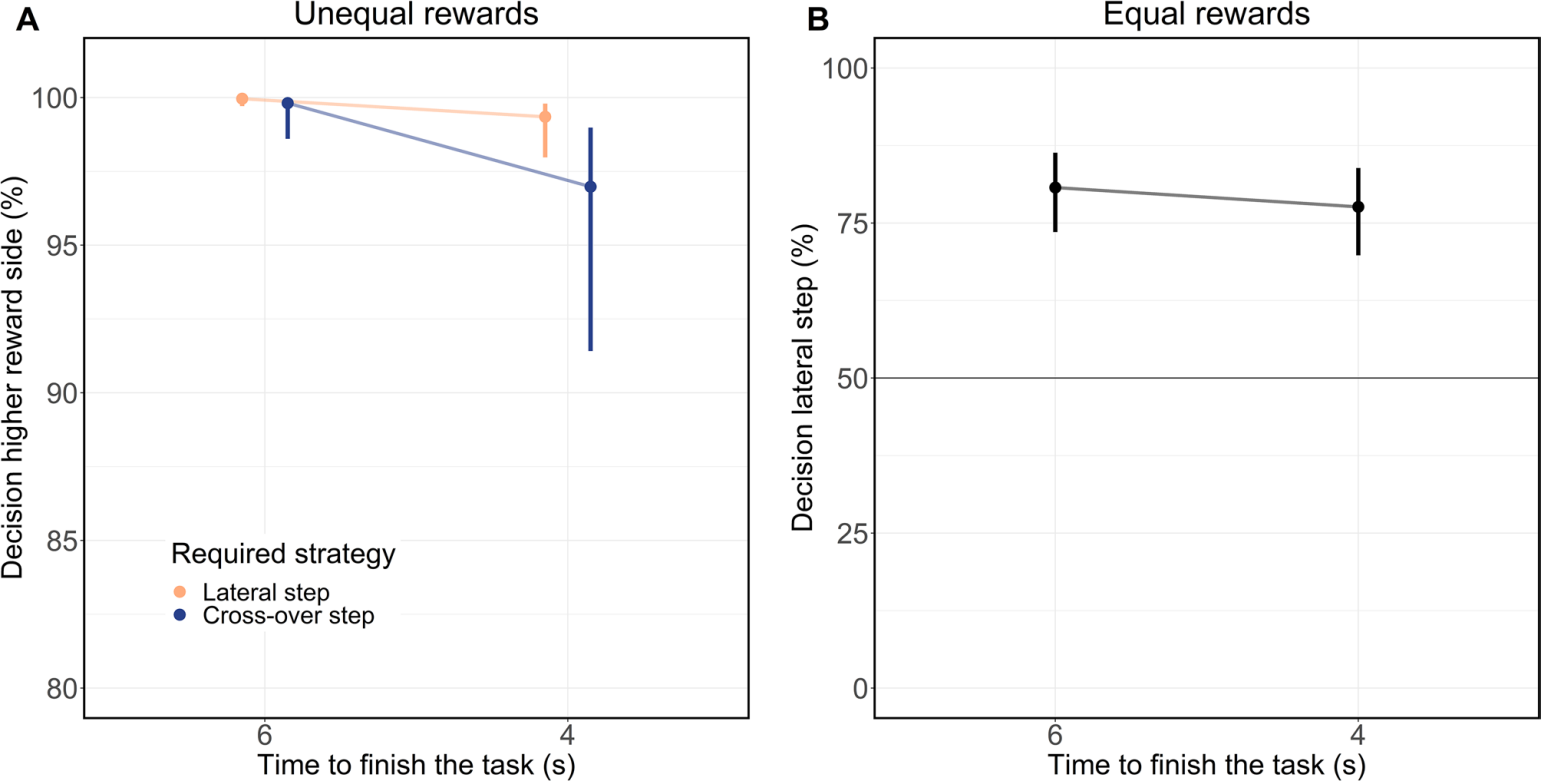
Influence of the step into the zone on decision-making in Exp. 2. Displayed are the estimates and 95% CI (Wald) of the respective GLMM. Note that the scale on the y-axis differs between both plots. (**A**) Effect of the step into the zone and time constraints on decisions for unequal rewards (e.g., 40/60 points left/right). If the step into the zone was incongruent to the side with higher reward (e.g., left step in the zone and higher rewards on the right side), this required a lateral stepping strategy to achieve higher rewards (orange circles). If the step into the zone was congruent to the side with higher reward (e.g., right step in the zone and higher rewards on the right side), this required a cross-over strategy to achieve higher rewards (blue circles). Participants walked more often to the side displaying lower rewards when a cross-over step was required, independent of the time to finish the task. (**B**) Probability to choose the side enabling a lateral step for equal rewards (50/50 points left/right) within both time conditions. A decision for a lateral step indicated that participants walked towards the incongruent side of the step in the zone. Participants went more frequently than chance level towards the side which enabled a lateral step independent of the time constraint.

For the unequal reward combinations (e.g., 60 points left vs. 40 points right, see Fig. 3A) participants less frequently walked towards the side with higher rewards when a cross-over step was dictated by the step into the zone (*χ*^2^ (1) = 6.55, *p* = 0.01, OR = 0.20, 95% CI [0.07, 0.63]).

Similarly, for the equal reward combination (50 points left/50 points right, see Fig. 3B), participants walked significantly more often than chance towards the side enabling a lateral step dictated by the step into the zone (Z = 7.41, p < 0.001, OR = 3.81, 95% CI [2.67, 5.43]). The different time constraints did not moderate the preference to walk towards the side enabling a lateral step, neither for unequal rewards (*χ*^2^ (1) = 0.01, *p* = 0.90, OR = 1.07, 95% CI [0.36, 3.17]) nor equal rewards (*χ*^2^ (1) = 0.78, *p* = 0.38, OR = 0.83, 95% CI [0.55, 1.24]). Additional model specifications and other estimations not related to the stepping strategy are presented in the SI (see Supplementary Tables S1 and S2).

To summarize, the results of Exp. 2 showed that the step into the zone and consequently the immediate body state at the time of reward information presentation influenced the value-based decision. Participants more frequently walked towards the side which afforded a lateral step and avoided the side of a costlier cross-over step even at the expense of receiving less reward. This result confirms that the dynamic action costs of the immediate body state are integrated into the decision, as proposed by action-based models^11^ and the embodied choice^10^ framework.

Despite showing that dynamic action costs are integrated into the decision, Exp. 2 was not designed to address the second main question of our study regarding the time course of action cost integration in value-based decisions. Therefore, in Exp. 3, next to the late presentation of reward information administered in Exp. 2, we systematically added two earlier time points of displaying the reward information during walking that potentially allowed participants to anticipate the final body state dictating the lateral or cross-over step. If the immediate body state at the time of reward presentation (and not the anticipated body state) affects the value-based decision, then the immediate body state should predict the final stepping direction independent of whether the immediate and the anticipated body state are congruent or incongruent, even when resulting in lower rewards at higher action costs.

### The anticipated rather than the immediate body state influenced decision-making

**Figure 4:**
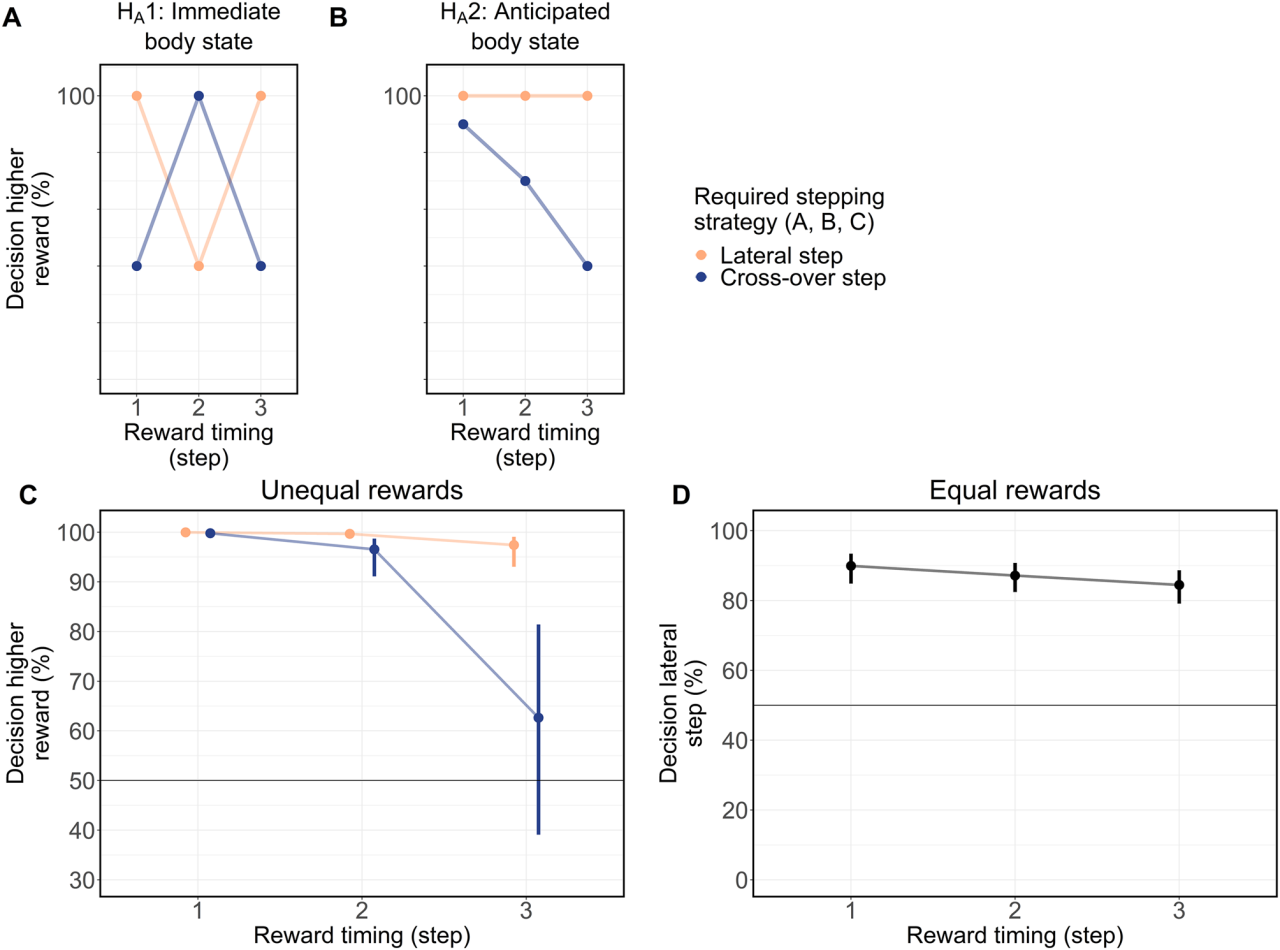
Alternative hypothesis and results for the influence of the stepping strategy and the timing of reward presentation on decision-making in Exp. 3. Reward timing (step) refers to the first (earliest presentation), second or third step after trial start. (**A**) Predicted results if the immediate step at reward presentation influences decisions. (**B**) Predicted results if the anticipated step into the zone influences decisions. (**C**) Estimates and 95% CI for decisions with unequal rewards (e.g., 60 vs. 40 points). Participants walked more often to the side displaying lower rewards when a cross-over step was required, independent of the time to finish the task. This effect descriptively increased when rewards were displayed later. (**D**) Estimates and 95% CI for decisions to walk to the side enabling a lateral step for equal rewards (50 vs. 50 points). Participants went more often than chance level towards the side which enabled a lateral step. The frequency to walk towards the side enabling a lateral step decreased when rewards were presented later.

To examine the time course of action cost integration in value-based decisions, in Exp. 3 the rewards were displayed at three different time points: the touch-down of the last step (identical to Exp. 2), the second-last step, and the third-last step before stepping into the zone. Because in Exp. 2 participants predominantly made four steps until reaching the zone, these time points corresponded to their first, second, and third step after initiating each trial (see “Methods” for how we ensured the four steps criterion). As a result, the different steps (i.e., immediate body state) at the time of reward presentation would differently affect lateral vs. cross-over stepping strategies. For instance, a third step making touch-down with left foot, thereby enacting a corresponding swing with the right leg for the final touch-down in the designated zone, would consequently lead to a lateral step to the left (see Exp. 2). In contrast, a second step making touch-down with right foot, thereby enacting a corresponding swing with left leg, would lead to a lateral step to the right. It follows that if the immediate body state accounted for a lateral vs. a cross-over stepping strategy, this would be different if predicted by the second step vs. the third step (see the previous example). However, if participants’ decisions were influenced by the anticipated body state when stepping into the zone, the direction of this effect should be independent of the time point and step (i.e., immediate body state) at which the rewards were presented. Note that such an anticipatory strategy may also be effectuated by means of stepping behavior adaptations, thereby reducing the influence of the body state on decisions. Consequently, Exp. 3 allowed us to differentiate between the integration of the dynamic action costs of the immediate vs. the anticipated body state (see Fig. 4A,B), and hence to scrutinize the time course of dynamic action cost integration.

As illustrated in Fig. 4C, for unequal reward combinations, independent of the time the reward information was presented, participants less frequently walked towards the side with higher rewards when a cross-over step was required by the final (anticipated) step into the zone (*χ*^2^ (1) = 24.61, *p* < 0.001, OR = 0.07, 95% CI [0.02–0.20]). Likewise, for the equal reward combination (see Fig. 4D), participants walked significantly more often than chance towards the side enabling a lateral step that was dictated by the anticipated step into the zone (*Z* = 10.96, *p* < 0.001, OR = 6.91, 95% CI [4.89, 9.76]). Together, these two findings support the hypothesis that the anticipated and not the immediate body state influenced decision-making (Fig. 4B).

**Figure 5:**
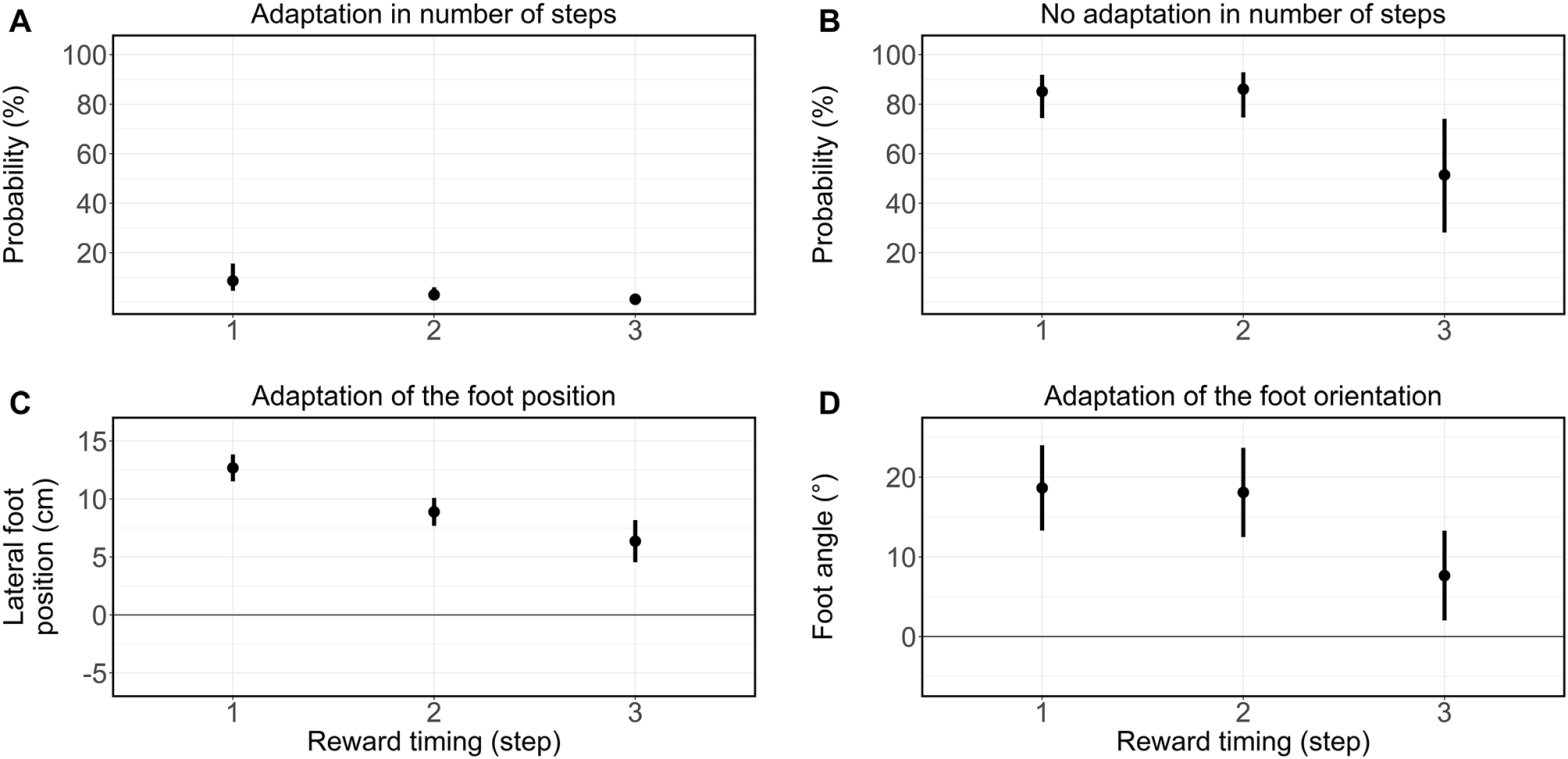
Adaptation of stepping behavior for different timings of the reward presentation. Illustrated are only trials in which participants received higher rewards and—to achieve those—the regular four steps would have led to a cross-over step. Displayed are the estimates and 95% CI for individual (generalized) linear mixed models. (**A**) Probability that participants adapted the number of steps (three or five steps instead of four) to change the step into the zone and enable a lateral stepping strategy when walking towards the side with a higher reward. The probability of adaptation decreased the later the rewards were shown. (**B**) Probability for cross-over steps when participants did not adapt their number of steps to walk towards the side with higher rewards. The frequency of cross-over steps decreased particularly between the reward presentation at the second and third steps. The probability of cross-over steps was notably higher compared to the probability of adaption of the number of steps to enable a lateral step [see (**A**)]. (**C**) Side independent lateral foot position (marker at the lateral malleolus) for the step into the designated zone. Only trials where the final step into the zone required a cross-over step were included [see (**B**)]. Positive values indicate a foot positioning towards the side participants finally walked to. (**D**) Side independent foot orientation for the step into the designated zone. Only trials where the final step into the zone required a cross-over step were included [see (**B**)]. Positive values indicate orientations towards the side participants finally walked to.

Given that, in addition, the interaction between reward presentation and required stepping strategy also almost attained significance for unequal reward combinations (*χ*^2^ (2) = 5.03, *p* = 0.08, first step vs. second step: *Z* = − 0.99, p = 0.32, OR = 0.58, 95% CI [0.20, 1.69], second step vs. third step: Z = − 1.72, p = 0.09, OR = 0.45, 95% CI [0.18, 1.12]), we argue that the effect of the anticipated body state on decision-making was likely effectuated by means of step adaptations (see Fig. 4C). To test this, in a subsequent step we analyzed whether participants (i) adapted the number of steps (see Fig. 5A,B) and (ii) the foot placement (location and orientation, see Fig. 5C,D) of the step into the zone^26^ when the rewards were displayed early. Additional model specifications and other estimations not related to the stepping strategy are presented in the SI (see Supplementary Tables S3 and S4).

### Participants adapted their stepping behavior when rewards were displayed early

As illustrated in Fig. 5A, participants indeed adapted the number of steps more frequently the earlier the rewards were presented (*χ*^2^ (2) = 27.19, p < 0.001). This was true for the difference between the second step and the first step (*Z* = − 4.12, p < 0.001, OR = 0.33, 95% CI [0.20, 0.56] as well as the third step and the second step (*Z* = − 2.69, p = 0.007, OR = 0.40, 95% CI [0.21, 0.78]).

However, given that participants seemingly preferred to not adapt the number of steps when going for the higher reward by maintaining a cross-over step (see Fig. 5B), we further analyzed whether foot placement adaptations when stepping in the designated zone facilitated this stepping strategy. Participants indeed placed the foot further to the side they decided to walk to the earlier the rewards were presented (*χ*^2^ (2) = 47.88, *p* < 0.001, first step vs. second step: *t* = − 9.86, p < 0.001, estimated difference = − 3.79 cm, 95% CI [− 4.55 cm to − 3.04 cm], second step vs. third step: *t* = − 3.11, p = 0.002, estimated difference = − 2.53 cm, 95% CI [− 4.12 cm to − 0.93 cm], see Fig. 5C). Additionally, they oriented (i.e. pointed) the foot further towards the side they decided to walk to when rewards were presented earlier than the latest time point, (*χ*^2^ (2) = 14.98, *p* < 0.001, first step vs. second step: *t* = − 0.40, p = 0.69, estimated difference = − 0.57°, 95% CI [− 3.32° to 2.18°], second step vs. third step: t = − 4.23, p < 0.001, estimated difference = − 10.43°, 95% CI [− 15.26° to − 5.60°], see Fig. 5D). Together, these results indicate that participants sometimes adapted the number of steps and far more often—when they did not adapt the number of steps—changed the foot placement (location and orientation) of the step into the zone when the rewards were displayed early.

To conclude, with earlier reward presentations participants adapted their stepping behavior to receive higher rewards. This provides additional (and perhaps more fine-grained) evidence for an effect of the anticipated body state on decision-making, thereby supporting the idea that dynamic actions costs affect the value-based decisions^10–13^.

## Discussion

The embodied choice framework^10^ and action-based models^11–13^ predict that action dynamics of concurrent movement are part of the decision process. To test this prediction, we first examined whether the action dynamics of walking, a complex whole-body motor behavior, affect value-based decision-making. In a second step, we scrutinized the time course of action cost integration.

Prior to addressing the first aim, we developed and validated our experimental paradigm by elucidating the preference for stepping strategies within sequential decisions (Exp. 1). In line with previous findings, we predicted a preference for the lateral rather than the cross-over stepping strategy^19–21^. Results confirmed this prediction, thereby indicating that cross-over steps are indeed costlier than lateral steps in our setup.

To address the first aim, Exp. 2 was then designed to investigate the integration of the immediate body state and associated action dynamics into the decision process by presenting the reward information so late that the immediate body state would necessarily dictate the subsequent lateral or a more costly cross-over step. Indeed, the immediate body state influenced the value-based decision: that is, participants walked more frequently towards the side which enabled a lateral step, and avoided the side of a costlier cross-over step even at the expense of receiving less reward. In keeping with research on reaching^17^, this finding seems to confirm that the dynamic action costs of the immediate body state are part of the decision process, thereby substantiating predictions of action-based models^11^ and the embodied choice framework^10^.

Subsequently, in Exp. 3 we replicated Exp. 2 and further aimed at scrutinizing the time course of action cost integration in value-based decisions by systematically manipulating the time points of displaying the reward information during walking. This manipulation allowed us to disentangle the influence of the immediate vs. the anticipated body state on decision-making. Results showed that, in contrast to research with passive movement on reaching^17^, the anticipated body state influenced decision-making: first, participants less frequently walked towards the side with higher rewards when a cross-over step was required by the final (anticipated) step into the zone independent of the time point of reward presentation. Second, in the case of walking towards the side with higher rewards, participants tended to adapt their action dynamics based on the (anticipated) body state that would finally facilitate this decision. This adaptation effect showed the earlier the rewards were presented.

Our findings provide first evidence that whole-body action dynamics affect value-based decisions, thereby on the one hand extending research on sequential decision-making in which cost and reward information are available before an action is initiated^3–7^. On the other hand, our findings also extend previous research that examined the effect of action dynamics on decision-making in (manual) reaching or finger tracking tasks^16–18^, by showing that the impact of dynamic action cost on value-based decision-making translates to whole-body motor behaviors such as walking.

While we were able to show that action dynamics and their associated costs affect value-based decisions, future research needs to identify and further specify the nature of action costs. We deem it likely that biomechanical costs^20^ as well as stability costs^19^ associated with walking play a major role in action dynamic integration in decision-making. It is also conceivable that other costs such as cognitive costs, including switching motor plans^27,28^, temporal discounting^29^ or weighting risks^30^ (see information about the time to finish and task success in the SI) may be involved.

Finally, Exp. 3 revealed that participants integrated the anticipated body state into their concurrent action planning and execution, thereby possibly reducing their action costs. More adaptation of action dynamics was observed the more time there was (i.e., the earlier the rewards were displayed) to implement the decision. The observed adaptation rules out that participants delayed their decision to the last step, thereby specifying the time course of action cost integration.

Next to revealing that adaptation was dependent on the time of reward presentation, Exp. 3 also showed that the effect of the anticipated body state on the decision trended to be dependent on the time of reward presentation. In other words, and as illustrated in Fig. 4C, this effect of the anticipated body state on decision-making diminished the earlier the reward information was presented, and hence the more time was given to adapt. We speculate that participants continuously update the anticipated body state and consequently have the opportunity to more effectively reduce the associated actions costs the earlier the reward information is provided.

There are some limitations that need to be addressed in future research. In our study, the effect of body state showed relatively large variations between participants for unequal rewards (see the standard deviation of the required stepping strategy in the Supplementary Table S1 and S3 and Supplementary Fig. S1B and S1C). We speculate that perhaps different levels of physical activity and/or motivational factors might explain part of the variance. In addition, it is noteworthy that some participants even demonstrated ceiling effects for unequal rewards, especially in Exp. 2 and with early reward presentations in Exp. 3. That is, some participants always walked towards the side with higher reward, and some participants almost exclusively walked towards the side with lower reward when a cross-over step was required. This generates no effect for participants with a ceiling effect and comparatively high odds ratios for the latter kind of participants. As we used a mixed model with participants as a random effect, this led to high shrinkage of the estimates for participants with comparatively high odds ratios towards the population estimate, thereby resulting in values close to 100% (see Figs. 3A and 4C). We suggest that the ceiling effects could arise because of our fixed level of difficulty between participants. To avoid ceiling effects in future studies, the difficulty of the task or cost difference of the body state could be individualized (e.g. varying time constraints for individuals, scaling the setup based on participants’ height, physical activity, or constraining the required step placement when making a directional change).

Finally, in our experiments, rewards were presented by means of points, and the motor behavior was constrained by stepping into a designated zone before bypassing the obstacle. It follows that future studies may look into different kinds of rewards and choices (e.g. monetary reward, subjective preferences for goods, performance-related choices in sports, perceptual decisions) and do so while putting fewer constraints on participants’ motor behavior. Likewise, there are a plethora of other factors that may moderate the subjective value of choices in daily behavior, such as the cultural embedding^31^, emotional states^32^, and age^33^. Therefore, we recommend examining these and other potentially moderating factors to test whether our results prove robust and generalizable to other commonplace real-life situations.

To conclude, here we provide initial evidence that whole-body action dynamics during ongoing movement affect value-based decision-making. This finding may generalize to many daily situations including when walking down the aisle in the candy section and deciding which snack to go for.

## Methods

### Participants

Participants were recruited via a mailing list of the psychology department, and billboard postings at the sports science department at the Friedrich Schiller University Jena. Participants were compensated with payment (10.00 €/hour) independent of their overall performance. Each participant attended only one experiment. We based our sample size on prior studies with decision-making as a binary outcome variable^3,16–18^. Participants provided written informed consent before experimentation. The study was carried out following institutional guidelines. All experiments were approved by the ethics committee of the Faculty of Social and Behavioral Sciences of the Friedrich Schiller University Jena. Table 1 provides demographic information about the sample used in Exp. 1 to Exp. 3.

**Table 1:** Demographic information. We used the Edinburgh Inventory^34^ to classify participants' handedness and the Lateral Preference Inventory^35^ for footedness. Additional analyses (available in the public depository online) indicated that neither footedness nor handedness shifted (i.e. affected) participants' overall side preference or effect of the body state in Exp. 2 and Exp. 3. *f *female, *m* male, *ri* right, *le* left, *n* no preference, *md* missing data.

	Exp. 1	Exp. 2	Exp. 3
Sex	16 f, 20 m	15 f, 22 m	19 f, 16 m
Age (mean ± SD)	21.8 ± 2.4 years	22.6 ± 2.5 years	22.5 ± 3.0 years
Handedness	31 ri, 4 le, 1 n	32 ri, 1 le, 0 n, 4 md	31 ri, 0 n, 4 le
Footedness	31 ri, 2 le, 3 n	30 ri, 2 le, 4 n, 1 md	27 ri, 2 le, 6 n

#### Experiment 1

Thirty-six healthy adults were recruited. All participants were included in the final data analysis.

#### Experiment 2

Forty-one subjects were recruited. Overall, four participants had to be excluded from further analyses. For two participants the reward signal was displayed too late in most trials because of a long stride. One participant was removed because the instruction was not properly understood. Another participant was removed because the same foot stepped in the designated zone in every trial, making a comparison between left and right impossible. The remaining thirty-seven participants were analyzed.

#### Experiment 3

Fifty-four participants attended Exp. 3. In contrast to the second experiment (see “Data analysis”), participants more frequently changed the number of steps in the neutral reward condition when the rewards were presented one step before the designated zone (19/54 participants). As it is not possible to predict the step into the designated zone when the number of steps varies in this chosen “baseline” condition, this subgroup was excluded from further analyses. Thirty-five participants remained.

### Apparatus and stimulus

Figure 1 displays the general setup and dimensions of the experiments. Dimensions from the start to the obstacle and targeted desks were derived from van der Wel and Rosenbaum^23^. On each desk (height = 0.73 m) a 22″ screen (Asus VW222U) was positioned for the visual display of reward and feedback after the trial. Each screen displayed numerical points in the center with a white font on a black background. In Exp. 1, rewards were displayed immediately after a trial was initiated, and before participants started walking. In Exp. 2 and Exp. 3, both monitors first alerted the participants to prepare for the upcoming trial by displaying the German word for ready (“Bereit”). Additionally, the displays indicated the starting position for the feet via two shifted zeros (i.e., a higher zero on the left indicated that the left foot had to be in front of the right foot before starting a trial and vice versa). After the trial was initiated, both monitors displayed a go signal in the form of a “+” in the center of the screen. The go signal was replaced by the point combination while participants were walking towards the obstacle. After completion of a trial, the temporal feedback of the trial was displayed below the reward feedback (i.e., awarded points).

A black protective grating was used as an obstacle (HWC-B34, height = 1.03 m). Black tape was used as a mark on the floor and on the desk to provide orientation for the start area, the designated zone in front of the obstacle, and the position of the hand to finish a trial (see Fig. 1). Gait behavior was recorded by a 3D infrared system (Prime 17W, Optitrack, Corvallis, US) with eleven cameras (120 Hz). Participants wore self-brought non-reflective running shoes during the experiment and a tight-fitting top for the placement of the reflective marker on the body.

### Procedure

After providing informed consent and demographic information, nine reflective markers (12 mm) were placed on the lateral malleolus, heel, between the first and second metatarsal head and dorsum of the hand on both body sides as well as the fifth lumbar vertebrae. Subsequently, participants were given instructions.

#### Experiment 1

Before a trial began both feet had to stand in parallel at the starting line (see Fig. 1). Participants initiated a trial by bringing their hands close together (i.e., clapping) and subsequently rewards were displayed for both sides. Participants were instructed to collect rewards and to pick a side before starting to walk towards the obstacle. They were further instructed that they had to step into the designated zone in front of the obstacle and bypass it to get to the desk on which the chosen reward was displayed. A trial was completed by touching a mark on the desk. If the participant had at least one foot in the designated zone during the trial, the chosen reward was displayed in green, otherwise in red. After the trial participants walked back to the starting line and began with the next trial. For the reward, nine different reward combinations (i.e., point combinations) could be displayed (left/right: 20/80, 30/70, 40/60, 45/55, 50/50, 55/45, 60/40, 70/30, 80/20). Both rewards always summed to 100, so that the reward on the left side could be inferred based on the reward on the right side and vice versa. Each participant began the experiment with five familiarization trials followed by 135 trials (9 reward conditions, each condition containing 15 trials). All trials were randomized within participants. Unintentionally, the randomization seed was not altered in the first experiment for most participants (31/36 participants), which means that the order of trials was random within but mostly the same between participants. The experiment lasted approximately 50–60 min.

#### Experiment 2

The procedure was similar compared to the first experiment, but at the start of the trial the starting positioning of the feet were predetermined and instructed, a time constraint to finish the task was added, and the rewards were displayed when participants were already close to the obstacle. Participants were instructed to get into the indicated starting position (left or right leg in front) before self-initiating a trial. At this time point, the timing of the trial started, and the go signal appeared. Participants were asked to walk towards the obstacle and were told that the reward combination would appear on their way to the designated zone in front of the obstacle. The goal was again to collect the reward by touching the mark on one of the desks within a time constraint (4 s or 6 s). The time conditions of 4 s and 6 s were based on the speed preferences observed in Exp. 1 (m = 4.9, sd = 0.6 s). 6 s was easily achievable for all subjects, while 4 s was faster compared to the preferred time in Exp. 1, thereby inducing time pressure. At the end of the trial, the reward changed color, and time feedback was displayed on the chosen side. If the time constraint was not met, the color was displayed red, and participants received no reward for this trial. If the foot at the touch-down (see “Data analysis” for the definition of touch down) was not completely positioned in the designated zone, the reward color was yellow and participants received the reward, but they were encouraged to make sure to fully step into the designated zone in future trials. If both conditions were met, the reward color was green, and points were awarded. After the feedback participants walked back to the starting position and began the next trial. The different reward combinations with a higher reward on one side had similar effects on the lateral decision in Exp. 1. Therefore, in Exp. 2 only five different reward combinations were displayed (left/right: 20/80, 60/40, 50/50, 60/40, 80/20). The experiment was divided into two blocks for the time conditions (4 s or 6 s to finish the task). The order of the blocks for the time conditions was counterbalanced across participants. Before each block 20 familiarization trials were performed, 10 without time evaluation and 10 with time evaluation. Each block consisted of 100 trials (5 reward combinations × 2 starting positions × 10 trials per condition). Overall, 240 trials were completed in one session of about 80–90 min. After the first block, participants had a 1-min break. Reward combinations and starting positions were randomized between trials.

#### Experiment 3

The procedure was almost the same as in Exp. 2. All trials were performed in the 4 s time constraint condition. The timing of the reward display was either after the first, second, or third touch-down (i.e., step making ground contact). Different reward combinations with a higher reward on one side had similar effects on the lateral decision in Exp. 2. Therefore, in Exp. 3 only three different reward combinations were displayed (left/right: 40/60, 50/50, 60/40). After the instruction, participants started with 18 familiarization trials, 9 without timing evaluation, and 9 with timing evaluation. The experimental session consisted of 180 trials (3 reward combinations × 3 timings of the reward × 2 starting positions × 10 trials per condition) and lasted around 60 min. After 90 trials participants had a 1-min break. All conditions were randomized between trials.

### Real-time analysis

To identify the start, the success of stepping in the designated zone, and the completion of a trial in real-time, the position of the tracked marker was streamed from Motive 2.1.1 (Optitrack software interface) with the NatNet SDK to a self-written written MATLAB 2018a script (The Mathworks, Inc., Natick, MA, USA). A trial started, when the distance between two markers in the expected hand area was below 15 cm. Additionally, in Exp. 2 and Exp. 3, the malleolus marker of the correct foot had to be 20 cm in front of the other foot. To prevent an early launch, the displacement of the calcaneus marker between two consecutive frames had to be below 2 mm when the trial was started by bringing the hands together.

The assignment of marker positions to body parts was achieved by utilizing the standardized starting position at the start of the trial. The positioning of hand markers was assumed to be in front of the L5 marker, the toe markers were in front of the heel marker, left body parts were more to the left, and so forth. The body-specific marker ID given by the Motive software was used for the assignment of markers for the rest of the trial. In rare cases, this ID changed because of the occlusion of a marker. When a relevant marker was missing because of a wrong assignment before reward feedback was displayed, the trial was repeated. To check if participants stepped into the designated zone and for the timing of the reward presentation in Exp. 2 and Exp. 3, the touch-down of every step was calculated as the maximal horizontal displacement of the heel marker of the swing foot and the malleolus marker of the stance limb^25^. To ensure only one maximum and touch down per step, after each maximum further analysis was skipped for 20 frames (0.167 s). In Exp. 2 we aimed to present the rewards one step before stepping into the designated zone. To do so, rewards were presented when the malleolus marker exceeded the 1.84 m distance from the starting line at touch-down. In Exp. 1, a step exceeding 1.84 m was in 97% the last step before stepping into the zone. In Exp. 3, we aimed to present rewards one step, two steps, or three steps before stepping into the zone. In Exp. 2 participants mainly made four steps with a 4 s time constraint. Therefore, rewards were displayed at the touch-down of the first, second, or third step in Exp. 3.

To test if the participant stepped into the designated zone, the position of the foot markers at every touch-down was compared with the area of the designated zone. All foot markers of the corresponding foot had to be in the designated zone. The trial was completed when a hand marker exceeded the horizontal position of the table marker at the beginning of the table and the hand marker was below 10 cm over the vertical height of the desk. The time between the start and end of the trial was used as time feedback after the trial.

We analyzed the lag of display for three pilot sessions. The frame of the touch-down was compared with the frame the display switched towards the reward stimulus with a synchronized reference camera. The lag between TD and the display of the rewards was consistent within one frame across trials and sessions (63 ± 7 ms, n = 32).

### Data analysis

Data preparation of kinematic data was accomplished using a self-written MATLAB 2018a code. The touch-down of every step was recalculated after the kinematic data were filtered at 12 Hz with a bidirectional fourth-order low-pass Butterworth filter. The foot stepping in the designated zone was identified as the first touch-down of a lateral malleolus marker into the designated zone (0.6 m in front of the marker at the obstacle, 0.3 cm towards both sides). The number of steps towards the obstacle was evaluated as the number of touch-downs until the step in the designated zone occurred. All touch-downs were double-checked by a second algorithm which was based on the relative velocity of both feet. As walking has a double stance phase with both feet on the floor, a step onto the ground should also be found by a minimum of the relative velocity of both malleoli markers. If there was an incongruence between both touch-down algorithms, the number of steps and step into the zone was visually checked and the algorithm with the correct values was picked. The positioning of the L5-marker in the y-axis at the end of the trial was used for assigning the lateral decision. Statistical analyses were performed with R^36^. All conditions were repeated measures over subjects. For the analyses of the dichotomous outcome of the lateral choice in each Experiment, a generalized linear mixed model (GLMM) was fitted with the glmer function of the lme4 package^37^. To account for the non-independence of repeated measurements, random intercepts and slopes for participants were entered as random effects. At first, the full random effect structure was fitted (random intercept, slope main effects, and all interactions). Because of convergence problems the full model was not acceptable for further analyses in most cases. If the model did not converge, we reduced the random effect structure by excluding random slopes each at a time, which were not relevant for our hypothesis, until the model converged^38–40^. Inference for the fixed effects was based on likelihood ratio tests between the model with and without the predictor variable. For the confidence intervals of the estimations, the Wald intervals were used. All tests were two-sided.

#### Experiment 1

The influence of the predictor “Lateral decision” (factor with 2 levels: left, right, simple contrast) on the outcome “Foot in the designated zone” (binary outcome: left, right) was analyzed by fitting a GLMM.

#### Experiment 2

Trials were omitted if the 1.84 m boundary for the reward display was not reached before stepping into the designated zone (overstepping, rewards were displayed too late). Two participants did this regularly (> 90% of trials) and were excluded from further analyses. Five individual trials were excluded because of problems with marker identification in the real-time analyses. After exclusion of trials and participants, a total of 7148 out of 8200 trials (i.e., 87.2%) entered statistical analyses.

In Exp. 2 five reward combinations were displayed. To reduce model complexity, we reduced the number of reward combinations to two levels, that is unequal reward combinations (e.g., 60/40 for the left/right side) and equal reward combination with no reward difference (50/50 for the left/right side). The unequal reward combinations were merged by mirroring the decision (left = right, right = left) and step into the zone (left = right, right = left) for reward combinations with more reward on the left side (80/20 and 60/40). After mirroring, the meaning of the “Decision” and “step in the zone” variable changed (decision: right = side with higher reward, left = side with lower reward; step in the zone: left = lateral stepping required to get towards the side with higher rewards, right = cross-over step required to get to the side with lower rewards).

For the statistical analysis of unequal rewards, the influence of the “Required stepping strategy” (factor with 2 levels: lateral or crossover step, simple contrast) and “Time constraint” (factor with 2 levels: 6 s and 4 s, simple contrast) and their interaction on the decision (binary outcome: higher reward, lower reward) was analyzed by fitting a GLMM. The requirement of a lateral step was defined as the step into the zone being incongruent to the side with higher reward (e.g., a left step into the zone and higher reward for the right target). The requirement of a cross-over step was defined as the step into the zone being congruent to the side with higher reward (e.g., a left step into the zone and higher reward for the left target).

For the statistical analysis of equal rewards, the influence of “Time constraint” (factor with 2 levels: 6 s and 4 s, simple contrast) on the decision to walk towards the side requiring a lateral step (binary outcome: yes, no) was analyzed by fitting a GLMM. For equal rewards requirement of a lateral step was defined as the step into the zone being incongruent to the side of the decision (e.g., a left step into the zone and walking towards the right target). The requirement of a cross-over step was defined as the step into the zone being congruent to the side of the decision (e.g., a left step into the zone and walking towards the left target).

#### Experiment 3

In Exp. 2, 31 out of 37 participants predominantly made four steps before stepping into the designated zone (mean = 98.8%, sd = 0.02%). In Exp. 3, the reward stimulus was supposed to be presented three steps, two steps, or one step before entering the designated zone. Therefore, we decided a priori to exclusively analyze participants who predominantly used four steps in the equal reward condition when the reward would be presented with the third step, like in Exp. 2. This criterion resulted in an unexpected exclusion of 19 out of 54 participants (based on k-means clustering with two clusters), who often did not use predominantly four steps before stepping into the designated zone (below 80% of the trials).

In Exp. 3 only three reward combinations were displayed. Like in Exp. 2, unequal reward combinations were merged. For the statistical analysis of unequal rewards, the influence of the “Required stepping strategy” (factor with 2 levels: lateral or crossover step, simple contrast), “Timing of reward presentation” (factor with 3 levels: 1. Step, 2. Step, 3. Step, sliding difference contrast) and their interaction on the decision (binary outcome: higher reward, lower reward) was analyzed by fitting a GLMM.

For the statistical analysis of equal rewards, the influence of “Timing of reward presentation” (factor with 3 levels: 1. Step, 2. Step, 3. Step, sliding difference contrast) on the decision to walk towards the side requiring a lateral step (binary outcome: yes, no) was analyzed by fitting a GLMM. The definition of the required stepping strategy was similar to Exp. 2, the only difference being that the expected step into the zone was used (given by the starting position and 4 steps before reaching the zone) and not the actual step into the zone.

Additionally, we analyzed adaptation strategies when participants starting position was in an unfavored body state (predicted cross-over step if participants would make the regular four steps) for getting towards the side the higher reward. First, participants could adapt their number of steps to change the body state when stepping into the designated zone, meaning that the step into the zone is not equal to the predicted step into the zone based on the starting position to make a lateral step towards the side with a higher reward. The influence of the “Timing of the reward presentation” (factor with 3 levels: 1. Step, 2. Step, 3. Step, sliding difference contrast on the binary outcome “Adaptation of the number of steps” (yes/no) was analyzed by a GLMM.

Second, they could make a crossover step and not adapt their stepping behavior to get to the side with a higher reward. For trials in which participants did a cross-over step the lateral positioning and orientation of the foot stepping into the zone were analyzed. For the lateral position, the malleolus marker of the foot stepping into the zone was used. The orientation was defined as the angle between the line of the global y-direction (in walking direction) and the vector spanning between the heel marker and the toe marker in the x–y-plane (lateral direction, walking direction). Foot position and orientation were analyzed with individual linear mixed models with the procedure used for GLMMs. Side-specific effects (left/right) were neutralized by merging over cross-over steps towards the left and right side and taking the negative for cross-oversteps towards the right side. The outcome position and angle (continuous scale) were predicted by the factor “Timing of reward presentation” (factor with 3 levels: 1. Step, 2. Step, 3. Step, sliding difference contrast).

## Supplementary Information

Supplementary Information.

## Data Availability

The data will be made available at: https://osf.io/2srwb/?view_only=218db3c5e58147a2973862550e7c5759.

## Code availability

Custom codes for the statistical analysis will be made available at: https://osf.io/2srwb/?view_only=218db3c5e58147a2973862550e7c5759.
