# Supplementary material for: Body dynamics of gait affect value-based decisions
Source: Sci Rep. 2021 Jun 4;11:11894. doi: 10.1038/s41598-021-91285-1 (PMC8178314; doi:10.1038/s41598-021-91285-1)
Supplement: Supplementary file 1 — Supplementary Information. [file 41598_2021_91285_MOESM1_ESM.pdf]

# Body dynamics of gait affect value-based decisions

Eric Grießbach[1*], Francesca Incagli[2], Oliver Herbort[2]

& Rouwen Cañal-Bruland[1*]

[1] Department for the Psychology of Human Movement and Sport, Friedrich Schiller University

Jena, Germany

[2] Department of Psychology, Julius-Maximilians-Universität Würzburg, Germany

*Correspondence concerning this article should be addressed to Eric Grießbach

(eric.griessbach@uni-jena.de) or Rouwen Cañal-Bruland (rouwen.canal.bruland@uni-jena.de).

# Supplementary Information

## Individual data for decision-making

The main article provides model estimations and 95% CI of the merged reward combinations (Fig 3 and Fig 4) without considerations of individual data. To give a more complete overview of the distribution of individual data before merging over rewards, scatterplots are provided for decision-making of Exp. 1 to Exp. 3 in Fig S1.

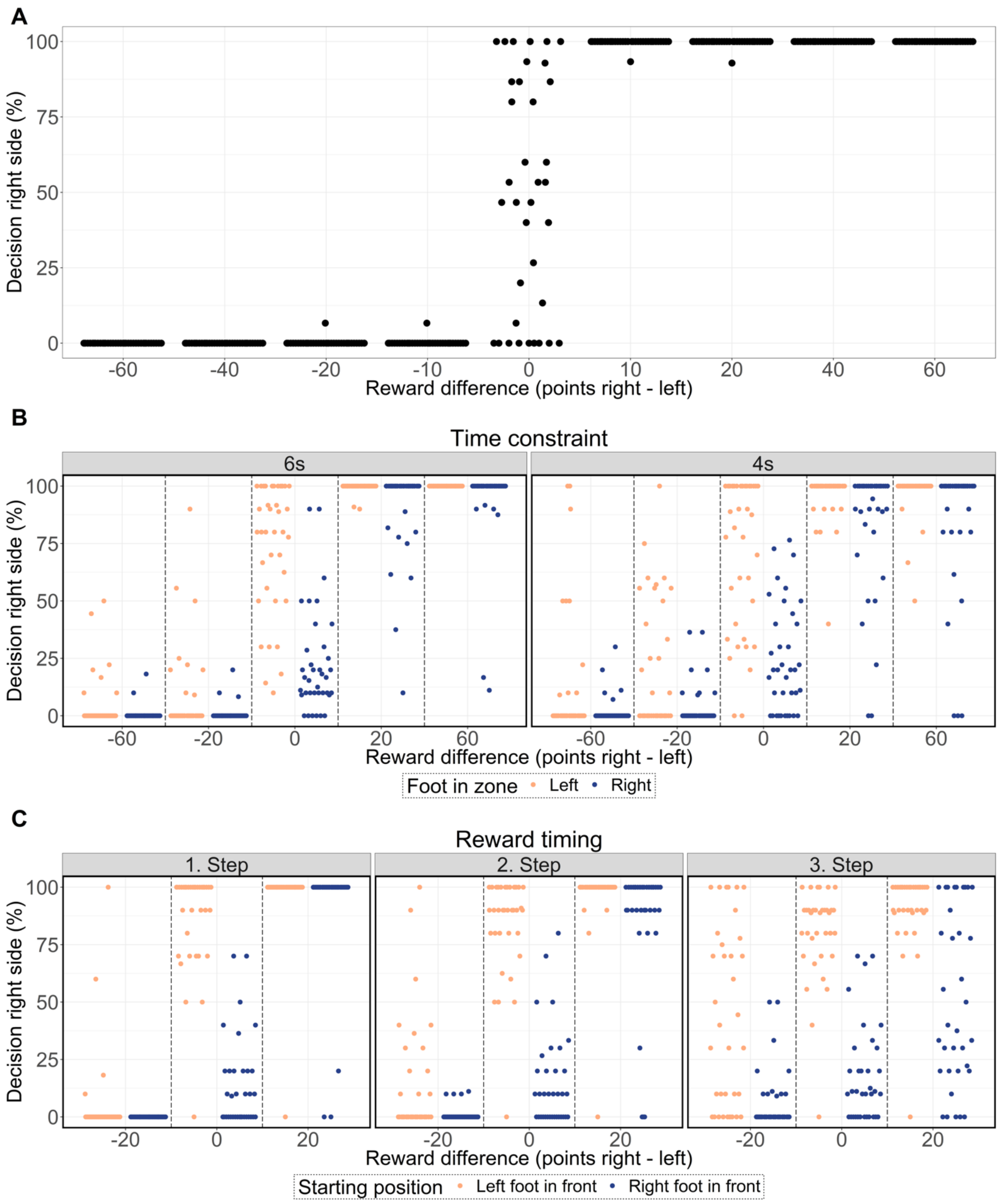


**Fig S1. Scatter plot of the individual data for decision-making in Exp. 1 to Exp. 3.** Dots display the probabilities for individual subjects and are jittered for better visual inspection. 0 % indicates that participants always walked towards the left side. Note that in some conditions participants almost always went to the side with higher rewards, resulting in the stacking of individual means at 0 or 100 %. A. Exp. 1. B. Exp. 2. A lateral stepping strategy was required when the decision and step into the zone were incongruent (e.g., left step into the zone and walking towards the right target). A cross-over stepping strategy was required when the decision and step into the zone were congruent. C. Exp. 3. The starting position corresponds to the step into the zone for the regular four steps participants observed in Exp. 2.

## Model specifications for decision-making in Exp. 2 and Exp. 3

The main text provided model terms and likelihood ratio tests relevant to our hypothesis. Further model specifications including additional model terms and the inclusion of random effects are specified in supplementary tables S1 to S4. These tables only provide Wald statistics (Z), and no likelihood ratio tests for the inference of non-hypothesis relevant terms. Note that odds ratios higher than one indicate that decisions were more likely towards the side with higher rewards (table S1 and S3) or the right side (table S2 and S4) and vice versa for odds ratio lower than 1. The defined contrast resulted in that the first-named level (e.g., for RSS: Lat in table S1 and S3) was always compared to the second-named level in the brackets (e.g., for RSS: Cross in table S1 and S3).

**Table S1. GLMM estimations for unequal rewards in Exp. 2.** The decision to walk towards the side with higher rewards (yes, no) was predicted by the factor "Required stepping strategy" (lateral or cross-over step), "Time constraint" (4 s or 6 s), and the interaction between both predictors. The random effect "Participant" with intercept and slope for the "Required stepping strategy" and "Time constraint" were included with correlation terms. OR = Odds ratio, RE = Random effect, TC = Time constraint, RSS = Required stepping strategy, Lat = Lateral step, Cross = Cross-over step.

| *Predictors* | *Log-Odds* | *OR* | *CI* | *Z* | *p* | *RE Std.* |
|---|---|---|---|---|---|---|
| Intercept | 5.66 | 152.05 | 91.31 to 908.67 | 9.66 | **<0.001** | 2.25 |
| TC (4 s vs. 6 s) | -2.83 | 0.06 | 0.01 to 0.43 | -2.80 | **0.005** | 3.63 |
| RSS (Lat vs. Cross) | -1.59 | 0.20 | 0.07 to 0.63 | -2.77 | **0.006** | 2.09 |
| TC (4 s vs. 6 s) : RSS (Lat vs. Cross) | 0.07 | 1.07 | 0.36 to 3.17 | 0.12 | 0.906 | - |

**Table S2. GLMM estimations for equal rewards in Exp. 2.** The decision to walk towards the side requiring a lateral step (yes, no) was predicted by the factor “Time constraint” (4 s or 6 s). The random effect “Participant” with intercept and slope for “Time constraint” were included with correlation term. OR = Odds ratio, RE = Random Effect, TC = Time constraint.

| *Predictors* | *Log-Odds* | *OR* | *CI* | *Z* | *p* | *RE Std* |
|---|---|---|---|---|---|---|
| Intercept | 1.34 | 3.81 | 2.67 to 5.43 | 7.41 | **<0.001** | 0.99 |
| TC (4 s vs. 6 s) | -0.19 | 0.83 | 0.55 to 1.24 | -0.91 | 0.362 | 0.84 |

**Table S3. GLMM estimations for unequal rewards in Exp. 3.** The decision to walk towards the side with higher rewards (yes, no) was predicted by the factor “Required stepping strategy” (lateral or cross-over step), “Timing of reward presentation” (1. step, 2. step, 3. step) and the interaction between both predictors. The random effect “Participant” with intercept and slope for “Required stepping strategy”, and the “Timing of reward presentation” were included without correlation terms. Note that in the main text for estimations and inference of the interaction term we used an additional model which included the interaction as a random effect, as suggested by[1]. Hence the values for the interaction deviate here. OR = Odds ratio, RE = Random effect, TC = Time constraint, RSS = Required stepping strategy, Lat = Lateral step, Cross = Cross-over step.

| *Predictors* | *Log-Odds* | *OR* | *CI* | *Z* | *p* | *RE Std.* |
|---|---|---|---|---|---|---|
| Intercept | 4.68 | 107.60 | 40.97 to 282.61 | 9.50 | **<0.001** | 2.21 |
| TR (2. step vs 1. step) | -2.89 | 0.06 | 0.01 to 0.25 | -3.75 | **<0.001** | 1.76 |
| TR (3. step vs 2. step) | -2.47 | 0.08 | 0.04 to 0.16 | -7.51 | **<0.001** | 1.18 |
| RSS (Lat vs. Cross) | -2.67 | 0.07 | 0.02 to 0.20 | -4.92 | **<0.001** | 2.42 |
| TR (2. step vs 1. step) : RSS (Lat vs Cross) | 0.09 | 1.09 | 0.26 to 4.53 | 0.12 | 0.905 | - |
| TR (3. step vs 2. step) : RSS (Lat vs Cross) | -0.70 | 0.50 | 0.21 to 1.17 | -1.59 | 0.111 | - |

**Table S4. GLMM estimations for equal rewards in Exp. 3.** The decision to walk towards the side requiring a lateral step (yes, no) was predicted by the factor “Timing of reward presentation” (1. step, 2. step, 3. step. The random effect “Participant” with intercept and slope for “Timing of reward presentation” of the first level (2. step vs. 1. step) were included without correlation term. OR = Odds ratio, RE = Random Effect, TS = Timing Reward.

| *Predictors* | *Log-Odds* | *OR* | *CI* | *Z* | *p* | *RE Std.* |
|---|---|---|---|---|---|---|
| Intercept | 1.93 | 6.91 | 4.89 to 9.76 | 10.96 | **<0.001** | 0.93 |
| TR (2. step vs 1. step) | -0.27 | 0.76 | 0.52 to 1.12 | -1.40 | 0.163 | 0.18 |
| TR (3. step vs 2. step) | -0.22 | 0.80 | 0.60 to 1.07 | -1.49 | 0.137 | - |

## Was decision-making in Exp. 1 sequential?

Exp. 1 served as a paradigm for sequential decision-making to determine the cost difference between the lateral step and the cross-over step. Therefore, the rewards were displayed before participants started walking. That is, cost and reward information were available before an action was initiated. However, participants were only instructed to choose before walking. If decisions were indeed sequential, we expected participants stepping behavior to reflect their decision (to implement the lateral stepping strategy observed in Fig 1) already at action initiation in the first step. Therefore, we ran additional analyses on the side of the first step and step length based on participants' choices. Results are illustrated in Fig S2 and indicate that most participants completed the decision before starting to walk.

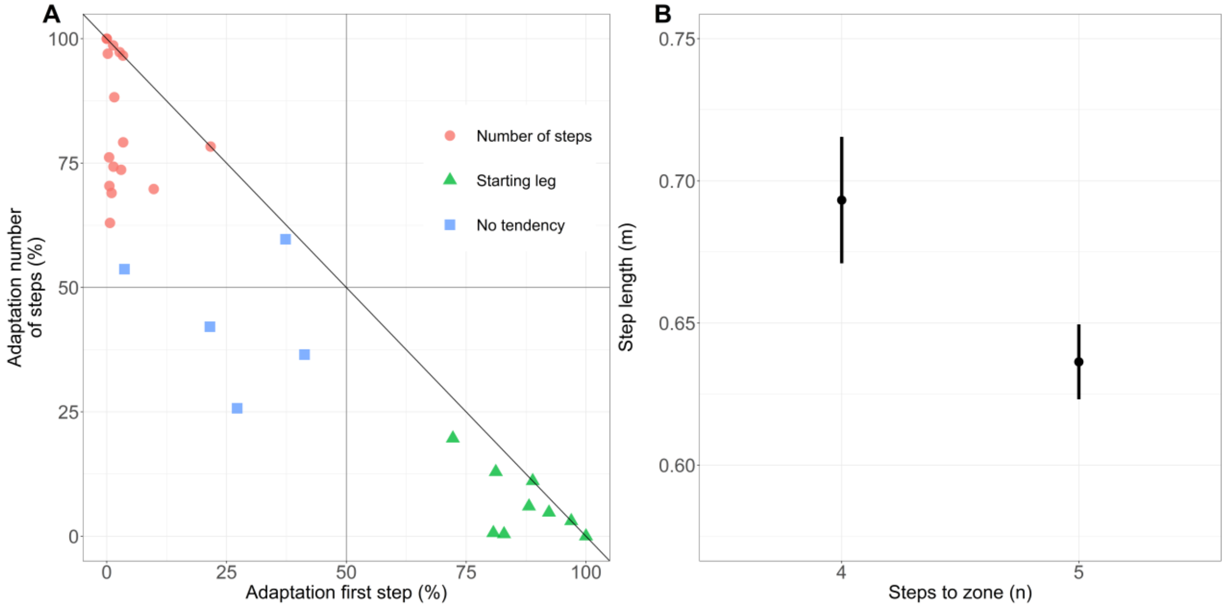


**Fig S2. Adaptation strategies of the first step to implement a lateral stepping strategy.** A. To enable a lateral step for both sides, participants had to switch the foot with which they stepped into the designated zone. This could be done by varying the stride length and therefore the number of steps (e.g., even to odd) or the side of the first step (e.g., left to right). Displayed is the percentual change in the number of steps (even or odd) and starting leg (left or right) between walking to the left compared to the right side. Values below the diagonal line are underspecified because there is no clear preference of stepping behavior for each side (e.g., 50 % even steps and 50 % right starting leg when going to the left and right side could be interchanged and would result in no difference for both variables). Each value is the probability for individual participants. Participants were classified into applying one of three strategies based on k-means-clustering. Adaptation of the first step provides evidence that action is selected after the specification of the decision (sequential decision-making). B. Analysis of the step length of the first step for participants which had no tendency or the tendency to change the number of steps. Displayed is the mean and 95% CI (within-subject). The step length of the first step was already shortened, suggesting that these participants also selected their action after the specification of the decision (sequential decision-making).

More specifically, to switch the foot in the designated zone, a change in the starting leg or a change in the number of steps could be performed. To evaluate adaptation strategies that enable a lateral step, the difference (in %) for both the starting leg and the number of steps (odd or even) between going to the left and right sides were calculated. One participant did not adapt the stepping behavior at all and was excluded from this analysis. Because data clusters were observed (see Fig S2A), the data were analyzed by k-means clustering (elbow method), to obtain the number of clusters and classify participants based on the style of adaptation. 9/35 participants already switched the side of the first step based on the decision to walk towards the left or right side, indicating that decision and action initiation was indeed sequential. For participants that did

not preferably adapt the side of the first step (26/35), we further analyzed the step length of the first step as the distance of both lateral malleolus marker at touch-down (see Fig S2B). One participant did not make 5 steps (but 3) and was excluded for the analyses of the step length. The step length of the first step was already shortened when adapting the number of steps from 4 to 5 steps ($t(25) = 6.35$, $p < 0.001$, $d_z = 1.25$, 95% CI [0.045 m, 0.088 m], see also Fig S2B). The early adaptation of the step length of the first step also indicates that decision and action initiation was indeed sequential.

### Information about the time to finish and task success

Exp. 2 and Exp. 3 involved a time constraint of 6 s (Exp. 2) or 4 s (Exp. 2 and Exp. 3). Participants received no reward when they finished the task too late. The time to receive rewards[2] and reward uncertainty are relevant factors of decision-making[3]. Therefore, the time to finish the task and task success was further analyzed.

As a manipulation check for the factor "Time constraint" in Exp. 2 we analyzed whether the time conditions influenced the time to finish the task. The average time to finish the task in the 6 s time condition was 4.40 s (sd = 0.36) and in the 4 s time condition 3.66 s (sd = 0.16). Differences in the time to finish the task were significant ($t(36) = 13.11$, $p < .001$, $d_z = 2.16$, 95 % CI = 0.62 to 0.85 s). In the 6 s time condition 0.1 % of trials were too late, in the 4 s time condition 7.2 % of trials were too late. A GLMM revealed that this difference as significant ($\chi^2$ (1) = 57.35, $p < 0.001$, OR = 0.02, 95 % CI = 0.01 to 0.07). This means that like expected participants finished slower and more often within the time limit in the 6 s time constraint.

Additionally, we compared the time to finish the task between the lateral stepping strategy and the cross-over stepping strategy in both time conditions with a repeated-measures

ANOVA. There was a significant interaction between time conditions and stepping strategy ($F$ = 31.82, $p < 0.001$, $\eta_p^2 = 0.006$). Separate dependent t-tests for both time constraints revealed that in both cases the time to finish the task was longer for cross-over steps and this difference increased in the 6 s time condition (4s: $t(35) = 4.68$, $p < 0.001$, $d_z = 0.78$, 95 % CI = -0.04 to -0.11, 6s: $t(35) = 7.28$, $p < 0.001$, $d_z = 1.21$, 95% CI = -0.11 to -0.20, p-values with Bonferroni correction). We also tested whether a cross-over step decreased the probability of getting towards the target in time and receiving the reward. In the 6 s time condition only 4/3673 trials were not in time (3 for lateral stepping behavior and 1 for the cross-over step). In the 4 s time constraint, trials with cross-over step were not significantly less frequent within the time constraint than trials with a lateral step ($\chi^2$ (1) = 1.04, $p = 0.31$, OR = 0.77, 95 % CI = 0.49 to 1.21), indicating that a cross-over step slowed participants but did not decrease the chance of receiving rewards.

Similarly, we also checked whether trials with a cross-over step compared to the lateral step involved a time cost in Exp. 3. The cross-over step was 0.07 s (95 % CI = 0.04 to 0.10 s) slower than the lateral step ($t(33) = 5.45$, p < 0.001, $d_z = 0.93$). The probability of reaching the reward in time decreased when doing a cross-over step compared to a lateral step ($\chi^2$ (2) = 8.92, $p = 0.002$, OR = 0.40, 95 % CI = 0.24 to 0.68), indicating that a cross-over step slowed participants and did decrease the chance of receiving rewards.

**References**


1 Barr, D. J. Random effects structure for testing interactions in linear mixed-effects models. *Front Psychol* **4**, 328 (2013).

2 Green, L., Myerson, J. & McFadden, E. Rate of temporal discounting decreases with amount of reward. *Mem Cognit* **25**, 715-723 (1997).

3 Mishra, S. Decision-Making Under Risk: Integrating Perspectives From Biology, Economics, and Psychology. *Pers Soc Psychol Rev* **18**, 280-307 (2014).
